# Broad thermal tolerance is negatively correlated with virulence in an opportunistic bacterial pathogen

**DOI:** 10.1111/eva.12673

**Published:** 2018-07-11

**Authors:** Roghaieh Ashrafi, Matthieu Bruneaux, Lotta‐Riina Sundberg, Katja Pulkkinen, Janne Valkonen, Tarmo Ketola

**Affiliations:** ^1^ Department of Biological and Environmental Science (and Nanoscience Center) Centre of Excellence in Biological Interactions University of Jyväskylä Jyväskylä Finland; ^2^ Department of Biological and Environmental Science University of Jyväskylä Jyväskylä Finland

**Keywords:** climate change, opportunistic pathogen, thermal performance curves, thermal tolerance, virulence

## Abstract

Predicting the effects of global increase in temperatures on disease virulence is challenging, especially for environmental opportunistic bacteria, because pathogen fitness may be differentially affected by temperature within and outside host environment. So far, there is very little empirical evidence on the connections between optimal temperature range and virulence in environmentally growing pathogens. Here, we explored whether the virulence of an environmentally growing opportunistic fish pathogen, *Flavobacterium columnare*, is malleable to evolutionary changes via correlated selection on thermal tolerance. To this end, we experimentally quantified the thermal performance curves (TPCs) for maximum biomass yield of 49 *F. columnare* isolates from eight different geographic locations in Finland over ten years (2003–2012). We also characterized virulence profiles of these strains in a zebra fish (*Danio rerio*) infection model. We show that virulence among the strains increased over the years, but thermal generalism, and in particular tolerance to higher temperatures, was negatively associated with virulence. Our data suggest that temperature has a strong effect on the pathogen genetic diversity and therefore presumably also on disease dynamics. However, the observed increase in frequency and severity of *F. columnare* epidemics over the last decade cannot be directly linked to bacterial evolution due to increased mean temperature, but is most likely associated with factors related to increased length of growing season, or other time‐dependent change in environment. Our study demonstrates that complex interactions between the host, the pathogen and the environment influence disease virulence of an environmentally growing opportunistic pathogen.

## 1 INTRODUCTION

Climate projections suggest that changing climate not only affects the average temperature but also the occurrence of extreme and variable temperatures (IPCC, 2007). Such changes in climate alter extinction risks, provoke range shifts and cause selection pressure to favour genotypes that are adapted to cope with these new environments (Heino, Virkkala, & Toivonen, 2009; Parmesan, 2006; Visser, 2008). Microbes, many of which have the capacity to be or become pathogens, are expected to adapt rapidly. Global warming may benefit many bacterial species, because they will face milder winter months resulting in greater overwintering success, increased numbers of generations and, thus, higher pathogen densities to damage hosts (Burdon & Chilvers, 1982; Coakley, Scherm, & Chakraborty, 1999). Environmentally growing opportunistic pathogens, in contrast to obligate (fully host‐dependent) pathogens, can utilize outside‐host resources, making them very sensitive to selection pressures outside the host (Brown, Cornforth, & Mideo, 2012). Therefore, predicting the effect of climate warming on environmental opportunistic bacteria with life cycles both outside and inside the host presents a particular challenge because pathogen fitness in both environments may be differentially affected by temperature (Harvell et al., 2002). Although the ability to stay alive in the environment, for example, as inactive spores, has been linked with high virulence (Day, 2002; Walther & Ewald, 2004), pathogens can also evolve towards a more benign virulence because investments in resource acquisition and defence in the outside environments can trade off with traits connected to virulence (Ketola et al., 2013; Mikonranta, Friman, & Laakso, 2012; Sturm et al., 2011; Sundberg, Kunttu, & Valtonen, 2014). Previous studies suggest that higher temperatures select genotypes that tolerate hotter temperatures, whereas fluctuations in temperature should select for more generalist genotypes with improved tolerance to extreme temperatures (Condon, Cooper, Yeaman, & Angilletta, 2014; Condon et al., 2015; Duncan, Fellous, Quillery, & Kaltz, 2011; Kassen, 2002; Ketola et al., 2013; Levins, 1968). Nevertheless, it has remained unclear how climate warming might affect growth parameters in environmentally growing opportunistic pathogens, and how this correlates with their potential to cause disease.

Understanding the selection pressures underlying the evolution of virulence in outside‐host environments is crucial in the current context of climate change, especially for diseases affecting world food production. *Flavobacterium columnare*, the aetiological agent of columnaris disease in farmed fish, is an opportunistic fish pathogen which severely impacts freshwater aquaculture worldwide (Bernardet & Grimont, 1989; Declercq, Haesebrouck, Van den Broeck, Bossier, & Decostere, 2013). Specifically, this bacterium can cause infections both in cold‐ and in warm‐water fish species such as carp, channel catfish, goldfish, eel, perch, tilapia, pike perch, rainbow trout, brown trout, salmon, tiger muskellunge and walleye (Anderson & Conroy, 1969; Schneck & Caslake, 2006; Shoemaker, Klesius, Lim, & Yildirim, 2003). *F. columnare* causes epidermal infections affecting gills, skin and fins of the fish, producing either acute or chronic infections, depending on the virulence and genetic group of the strain, as well as on environmental and host‐related factors (Declercq et al., 2013). The temperature range in which it can grow actively is approximately 15 to 35°C (Declercq et al., 2013). Previous work on this bacterium and a number of other virulent pathogens in the context of global warming has focused mainly on long‐term empirical data examining the relationship between mean ambient temperature and disease prevalence (Karvonen, Rintamäki, Jokela, & Valtonen, 2010; Pulkkinen et al., 2010). As both open and flow‐through aquaculture systems are connected to natural water bodies, it can be expected that changes in ambient water temperatures strongly affect farming conditions. Analysis of more than 20 years’ worth of data has showed a significant positive effect of mean water temperature on the prevalence of columnaris disease at two fish farms (Karvonen et al., 2010). At the same time, the data point to an increase in virulence of this bacterium in fish farms over the years (Pulkkinen et al., 2010), which might have happened due to selection for certain genotypes of the bacterium (Sundberg et al., 2016). However, it is still unclear whether climate change will impact the thermal performance of this bacterium in the long term by selecting more thermotolerant strains and whether such changes may have any effect on bacterial virulence. This is important information for regions where climate change is expected to be most severe, such as Finland where average annual temperature is predicted to rise nearly twice as fast as the average temperature for the whole globe (Ruosteenoja, Jylhä, & Kämäräinen, 2016).

**Figure 1:**
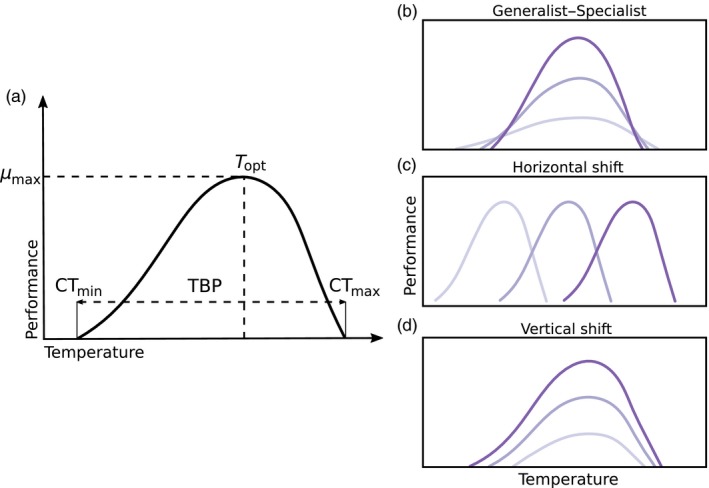
Hypothetical thermal performance curves with the commonly used descriptors of a performance curve (a) Main descriptors of performance. Patterns of variation for nonlinear thermal performance curves: (b) generalist–specialist, (c) horizontal shift, (d) vertical shift. *μ*_max_: maximum performance; *T*_opt_: optimal temperature; CT_min_: lower critical temperature; CT_max_: upper critical temperature; TPB: thermal performance breadth (redrawn from Kingsolver, Izem, & Ragland, 2004)

Thermal tolerance is usually depicted via thermal performance curves (TPC) composed from the measured performance of a genotype in different thermal environments. Assuming that thermal performance curves obtained from measurements taken in constant environments can be used to predict how genotypes survive under fluctuations (Huey, Berrigan, Gilchrist, & Herron, 1999; Ketola & Kristensen, 2017; Ketola & Saarinen, 2015; Sinclair et al., 2016), adaptation to fluctuating environments could occur either via overall elevated TPC or via broadened TPC (Ketola et al., 2013; Levins, 1968; Scheiner & Yampolsky, 1998). The key ecophysiological parameters that characterize thermal performance curves are the critical thermal thresholds which represent the lower (CT_min_) and upper (CT_max_) temperatures at which performance (e.g., growth or biomass yield of bacteria) is zero, the optimum temperature (*T*_opt_) at which performance is maximal, and the maximum value of performance itself (*μ*_max_). Ecological and evolutionary physiologists have proposed three directions or modes for changes in TPCs: in the width (or breadth; also called generalist‐specialist trade‐off), in the position of the Topt (through a horizontal shift of the curve; also called hotter‐colder mode) and in the height (through a vertical shift of the curve; also called faster‐slower mode) of the curve (Angilletta, 2009; Izem & Kingsolver, 2005; Kingsolver, Ragland, & Diamond, 2009; Figure 1). In addition to these parameters, variation in TPC can also be characterized using principal component analysis (PCA) on growth performances from different temperatures to identify the main patterns of performance variation among the genotypes (Huey & Kingsolver, 1989; Izem & Kingsolver, 2005).

In this study, we measured bacterial growth at five different temperatures (spanning from 17 to 32°C which matches typical summer growth season in Finland and in the near future) to characterize the temperature dependence of maximum biomass yield in 49 *F. columnare* isolates collected from Finland during the period 2003–2012. Based on this data, we examined (a) variation of thermal performance among isolates using two alternative approaches including estimation of TPC parameters for each strain and application of principal component analysis (PCA) on maximum yields from different temperatures and (b) the link between thermal performance and bacterial virulence, using virulence data measured in a zebra fish (*Danio rerio*) infection model. We showed that Finnish isolates differed in maximum yield and limits of thermal range and that their higher tolerance to high temperatures was linked to lowered virulence.

## 2 MATERIAL AND METHODS

### 2.1 *F. columnare* strains and culture conditions

We used 49 Finnish *F. columnare* isolates for which genotypes were previously determined by the conventional multilocus sequence typing (MLST) method using six loci (Ashrafi, Pulkkinen, Sundberg, Pekkala, & Ketola, 2015) (Supporting information Table S1). *F. columnare* are assigned into five genomovar groups using 16S rDNA restriction fragment length polymorphism analysis including genomovar I, II, II‐B, III and I/II (LaFrentz, Waldbieser, Welch, & Shoemaker, 2014; Triyanto & Wakabayashi, 1999). All strains belonging to genomovar I which has been characterized by low‐temperature tolerance (Suomalainen, Kunttu, Valtonen, Hirvela‐Koski, & Tiirola, 2006) were originally isolated from eight fish farms, from both northern (65°N) and southern (62°N) parts of Finland (Supporting information Table S1), from fish (mostly salmonids such as Atlantic salmon, brown trout or rainbow trout) or from tank water using standard culture methods with modified Shieh medium (Song, Fryer, & Rohovec, 1988), modified Shieh medium supplemented with tobramycin (Decostere, Haesebrouck, & Devriese, 1997) or AO agar (Anaker & Ordal, 1959).

### 2.2 Thermal performance measurements

Bacterial isolates were grown overnight in modified Shieh medium under constant agitation (120 rpm) in room temperature and further subcultured to fresh medium in ratio of 1:10 for another 16–18 hr under the same conditions. Sterile 15‐ml tubes containing 5.5 ml of bacterial culture were centrifuged for 5 min in 4°C at 3,500 *g*, after which the supernatant was discarded. 240 μl of concentrated bacterial culture was mixed with 60 μl of 10% of glycerol and 10% of foetal calf serum mixture on 100‐well Bioscreen C^®^ plate in a randomized order and stored at −80°C. Prior to growth measurements, bacterial isolates were inoculated to a new Bioscreen plate containing 400 μl fresh modified Shieh medium in each well directly from the frozen Bioscreen plate using heat‐sterilized cryo‐replicator (Enzyscreen B.V., Haarlem, the Netherlands (Duetz et al., 2000)). After 24‐hr incubation at 25°C, inoculums of 40 μl of individual bacterial strains from these precultures were distributed into a Bioscreen plate containing 400 μl of fresh modified Shieh medium in each well for the growth measurements. Growth experiments were run simultaneously in duplicate in two 100‐well plates in a Bioscreen C spectrophotometer (Oy Growth Curves Ab Ltd, Finland) over 2–8 days depending on the experimental temperature, at five different temperatures (17°, 22°, 24°, 29° and 32°C). The bacteria were cultured without shaking, and optical density (OD) measurements were performed at 5‐min intervals (absorbance at 600 nm). The growth curves were analysed as described in Ketola et al. (2013) to estimate maximum growth rate and maximum biomass yield. Maximum yield is estimated from the plateau phase of a growth curve while maximum growth rate is estimated from its early phase and is thus influenced by the potentially large relative noise in the OD measurement during this phase. Consequently, we chose to use maximum yield as a robust measure of strain performance at a given temperature (two measurements per temperature per strain). For each temperature, the repeatability of maximum yield measurement (intraclass correlation coefficient (ICC)) was defined as *R* = *V*_G_/(*V*_G_ + *V*_R_), where *V*_G_ is the variance among strains and *V*_R_ is the variance within strains (Sokal & Rohlf, 1995; Wolak, Fairbairn, & Paulsen, 2012). Repeatability was calculated using the rptR package for R (Stoffel, Nakagawa, & Schielzeth, 2017).

Two alternative approaches were used to analyse the thermal performance data: (a) for each strain, curve fitting using all maximum yield values followed by single‐point estimation of TPC parameters (i.e., one value for CT_min_, CT_max_, *T*_opt_ and *μ*_max_ per strain) and (b) principal component analysis (PCA) on the maximum yield values averaged per temperature for each strain.

### 2.3 Thermal performance curve fitting and parameter estimation

We used the TableCurve 2D software (version 5.01; Systat Software Inc., 2002) to select a set of candidate equations to describe the relationship between yield and temperature. Using data from a subset of experimental strains, all available equations described by functions with two or three terms plus an intercept (i.e., 1960 equations of all 3,665 equations available in the software library) were fitted and the resulting fits with large *R* ^2^ values were visually inspected. Candidate equations were selected based on the fulfilment of the following criteria to ensure a biologically meaningful fit: (a) “bell‐shaped” curve with maximum yield occurring within the experimental thermal range, (b) mostly concave curve (i.e., curves with several and clear local maximums in the experimental thermal range were discarded, but slight bumps were allowed), (c) extrapolation outside the experimental thermal range predicted decreased performance (i.e., the behaviour of the curve outside the experimental thermal range was consistent with biological expectations). In the end, the following six equations were chosen as candidates for a plausible model of the relationship between temperature (*x*) and performance (*y*):

(1) $$y=a+b\cdot x^{2}\cdot \log\left(x\right)+c\cdot x^{3}$$

(2) $$y=a+b\cdot x+c\cdot x^{2}$$

(3) $$y=a+b\cdot x^{\frac{3}{2}}+c\cdot x^{2}$$

(4) $$y=\frac{1}{a+b\cdot \exp\left(x\right)+c\cdot \exp\left(-x\right)}$$

(5) $$y=a+b\cdot x+c\cdot \log{\left(x\right)}^{2}+d\cdot \sqrt{x}$$

(6) $$y=a+b\cdot \log{\left(x\right)}^{2}+c\cdot \log\left(x\right)+\frac{d\cdot \log(x)}{x},$$

where *a*,* b*,* c* and *d* are strain‐specific curve parameters.

For each strain, a weighted‐average thermal performance curve was built after fitting those six candidate equations, where AIC values were used to calculate a strain‐specific weight for each of the six equations according to the formula:

$$w_{i}=\frac{e^{-\left(\Delta \text{AIC}\right)i/2}}{\sum_{1\leq j\leq 6}e^{-\left(\Delta \text{AIC}\right)j/2}},$$

where *w*_*i*_ is the weight assigned to the *i*th equation and (△AIC)_*i*_ is the difference between the AIC of the *i*th equation and the lowest AIC among the six equations for this strain. While acknowledging that our procedure for the selection of candidate equations might introduce some subjectivity in the choice of candidate curves, keeping six different candidate equations and producing a weighted‐average model based on their AIC values allowed for a variety of shapes in the final fitted curves with an overall good quality of fit, as shown in Supporting information Figure S1.

The obtained average thermal performance curves were used to determine maximum performance *μ*_max_ and optimal temperature *T*_opt_. We decided not to extrapolate unreasonably the thermal performance curves to determine CT_min_ and CT_high_ values, but instead chose to estimate thermal ranges by calculating for each strain the temperatures at which its TPC reached half its maximum performance, hereafter CT_50/low_ and CT_50/high_. Growth at lower temperatures falls gradually and the growth in this species is already unmeasurable at 15°C, causing estimation inaccuracy in curve fitting and in estimating CT_min_. Thermal performance breadth (TPB) was defined as the difference between the estimated CT_50/high_ and CT_50/low_. A visual inspection of the fitted curves was performed to remove CT_50/low_ (for 8 strains) and *T*_opt_ values (for three strains) which were unreliable given the shape of the fit for a particular strain (e.g., very flat plateau at *μ*_max_, unreliable extrapolation for CT_50/low_), resulting in 41 strains with all TPC parameters.

### 2.4 PCA on yield measurements

As PCA is sensitive to outlier data points departing from normal distributions, we visually inspected normal quantile–quantile plots of the yield data to identify and remove three outliers of 49 strains prior to PCA. PCA was performed using the covariance matrix of maximum yields in five temperatures. Although outliers were removed to minimize the possibility of exerting undue influence on the PCA by their departure from normality, these data point were otherwise biologically meaningful. Therefore, the coordinates of all 49 strains along each principal component (PC) were calculated based on the PCA loadings obtained from the subset of 46 strains. To make sure that the results did not depend on the specific treatment of outliers, we rerun all downstream calculations without the outliers, with no change in main results.

To facilitate the biological interpretation of the patterns of variation described by each PC, we predicted the TPC of hypothetical strains located at the extreme boundaries of the 95% range of the coordinates of experimental strains along each PC using the inverse of the PCA matrix.

### 2.5 Virulence assay

A virulence experiment was conducted according to the Finnish Act on the Use of Animals for Experimental Purposes, under permission ESAVI‐2010‐05569/Ym‐23 granted for L‐RS by the National Animal Experiment Board at the Regional State Administrative Agency for Southern Finland. Virulence of the 49 bacterial strains was assessed in an experiment using zebra fish (*Danio rerio*). The fish were infected using bacterial cultures grown overnight in fresh modified Shieh medium and adjusted at 4 × 10^5^ colony‐forming units (CFU)/ml. Ten fish per bacterial strain were individually challenged in 500 ml of water by adding 500 μl of adjusted bacterial culture directly into the experimental aquaria. The water temperature was maintained at 25°C during the experiment, which is close to the mean *T*_opt_ of the strains used. Aquaria containing fish were randomly placed on shelves in the experimental room to avoid systematic differences between aquaria. This infection method has been shown to produce a rapid onset of disease in fish, bringing out strain differences (Kinnula, Mappes, Valkonen, Pulkkinen, & Sundberg, 2017; Laanto, Penttinen, Bamford, & Sundberg, 2014). As a control, 10 fish were individually exposed to 500 μl of sterile modified Shieh medium. Disease signs and fish morbidity were monitored at 2‐hr intervals for 97 hr. Morbid fish that had lost their natural swimming buoyancy, and which did not respond to external stimuli, were considered dead and removed from the experiment and euthanatized by cutting the spinal cord to avoid the suffering of the fish. Although rainbow trout and other salmonids are the natural hosts of *F. columnare* in Finland, we used zebra fish as an experimental model for bacterial virulence in this study: Previous study has demonstrated that zebra fish and rainbow trout respond to bacterial doses and strains qualitatively similarly (Kinnula, Mappes, Valkonen, & Sundberg, 2015; Kinnula et al., 2017), allowing for reasonable extrapolation of virulence experiment results across hosts. In addition, rainbow trout is a cold‐adapted species, which makes it difficult to handle in laboratory conditions, while zebra fish is a well‐studied model organism available all year round and sharing the temperature optimum of *F. columnare*, making it a more practical model organism for this study.

### 2.6 Statistical analyses of thermal performance data

The effects of MLST (multilocus sequence typing) genotype group (categorical variable, four levels), year of strain isolation (continuous variable) and geographical location (categorical variable, two levels: Northern and southern Finland) on thermal performance were assessed using model selection starting from a full linear model specified as:

$$\begin{array}{cc} \text{Performance}=\; & \text{intercept}+\beta_{g}\cdot \text{Group}\\ & +\beta_{y}\cdot \text{Year}+\beta_{1}\cdot \text{Location}+\text{residuals} \end{array}$$

where performance was either one of the thermal performance curve parameters estimated from curve fitting (*μ*_max_, *T*_opt_, CT_50/low_, CT_50/high_ or TPB) or coordinates along one of the principal components of interest (PC1, PC2 or PC3). No interaction between Group and Year or Group and Location was included in the starting model due to the imbalanced distribution of strains from different MLST genotype groups across the years or across the geographical range of our study. Model selection was performed iteratively: At each step, variables were dropped one at a time and the significance of the change in fit for each dropped variable was tested using a chi‐square test (function *drop1* in R). If the highest *p*‐value for significance of change in fit was >0.10, the corresponding variable was dropped from the model and the next selection step was performed; otherwise model selection was stopped.

### 2.7 Statistical analyses of virulence data

As the vast majority of death events occurred early in the virulence assay, virulence data were analysed by considering fish survival as a binary variable (death/survival). The effects of explanatory variables on fish death were estimated using generalized linear mixed models (binomial family) with a logit link function and using strain identity as a random factor. Two full models differing in how they incorporated thermal performance as an explanatory variable (using either (1) PCs or (2) TPC parameters) were used as starting models. The fixed effects used in those two initial models were:

1. MLST genotype, year, location, PC1, PC2 and PC3 (49 strains)
2. MLST genotype, year, location, *μ*_max_, *T*_opt_ and CT_50/high_ (46 strains)

**Figure 2:**
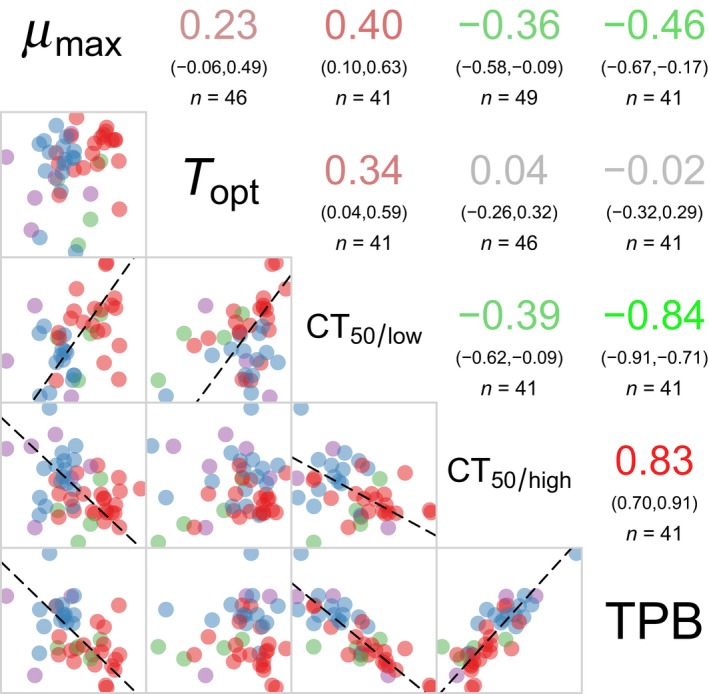
Correlogram for thermal performance parameter estimates. Upper triangle, Pearson's product moment correlation coefficients between pairs of variables. The numbers in brackets indicate the 95% confidence interval; the N value is the number of strains available to calculate the correlation coefficient. Lower triangle, scatter plot between pairs of variables. When the Pearson's product moment correlation coefficient is significant (*p*‐value <0.05), a dashed line indicates the ranked major axis. For the upper triangle, the colour coding on a green to red scale matches the correlation coefficient value (−1, green; 0, grey; +1; red). For the lower triangle, colours match multilocus sequence typing (MLST) genotype: red, purple, green and blue are for genotypes C, E, G and A&H, respectively. *μ*_max_, maximum biomass yield; *T*_opt_, optimal temperature; CT_50/low_ and CT_50/high_, lower and upper critical temperatures for which performance is half of *μ*_max_; TPB, thermal performance breadth

CT_50/low_ and TPB were not included in the full model (2) due to co‐linearity with CT_50/high_ (Figure 2).

Models were fitted using the glmer function from the lme4 package in R. Model selection was performed starting from each of the full models and testing the effect of removing one variable out at a time, and testing for the significance of the change in fit with a likelihood‐ratio test (function *drop1* in R). At each step, the variable with the highest *p*‐value for the significance of change in fit was dropped if this *p*‐value was >0.10. We used the DHARMa package in R (Hartig, 2016) to assess the correctness of the residuals.

## 3 RESULTS

### 3.1 Correlations between thermal performance curve parameters

Repeatability of yield measurements was reasonable between 17 and 29°C, but was lower for the highest temperature, close to maximum tolerable temperature (*R*_17°C_ = 0.808, *R*_22°C_ = 0.927, *R*_24°C_ = 0.747, *R*_29°C_ = 0.798, *R*_32°C_ = 0.595). TPC parameters were estimated from the AIC‐weighted‐average curves for each of the 49 strains. Due to uncertainty in estimated values for some fits, *T*_opt_ values were calculated for 46 strains, and CT_50/low_ values for 41 strains (Supporting information Figure S1, Supporting information Table S1). A correlogram was built to explore pairwise correlations between TPC parameters (Figure 2). CT_50/low_ and CT_50/high_ were negatively correlated, and thus, an increase in cold tolerance (i.e., decrease in CT_50/low_ value) was correlated with an increase in heat tolerance (i.e., increase in CT_50/high_ value), suggesting variation along a gradient of narrow to wide thermal range. *T*_opt_ was positively correlated with CT_50/low_ but not with CT_50/high_, which reflects a horizontal shift of the left‐hand part of the TPC while maximum thermal tolerance is more constrained. Finally, *μ*_max_ was positively correlated with CT_50/low_ and negatively correlated with CT_50/high_ and TPB; this might reflect a trade‐off between increased tolerance to a larger range of temperatures and higher maximum performance.

### 3.2 Principal components describing variation in thermal performance

**Figure 3:**
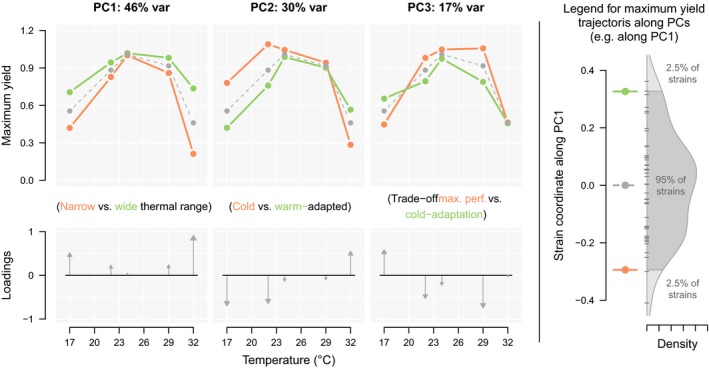
PCA results and their interpretation in terms of TPC patterns. Upper panels, prediction of TPC variation patterns along each of the first three PCs. Grey dashed line, average performance curve of the 49 strains used in this study. Orange and green lines, performance curves of hypothetical strains with coordinates at the lower and upper 95% quantiles, respectively, along each PC, as depicted in the explicative panel on the right. Lower panels, loadings for each original temperature on the first three PCs

We selected the first three principal components (PCs), which accounted for 93% of the variability of the yield measurements at 17, 22, 24, 29 and 32°C for 46 strains (Figure 3, Supporting information Figure S2 and Table S2). PC1 (46% of variation) describes correlated changes in performance at the extreme temperatures (17 and 32°C) while performance at the optimum temperature remains unchanged. PC1 thus mostly describes a gradient from narrow to wide thermal range. PC2 (30% of variation) is characterized by a negative correlation between performance in cold and warm temperatures (17°C, 22°C vs 32°C) and PC2 can be seen as a cold adaptation/warm adaptation axis. PC3 (17% of variation) corresponds to a negative correlation between performances in the coldest temperature (17°C) and in medium temperatures around the optimal temperature (22–29°C) and could to some extent be seen as a trade‐off between cold adaptation and optimum performance.

### 3.3 Determinants of thermal performance

The MLST genotype affected all calculated thermal performance parameters (*μ*_max_, *T*_opt_, CT_50/low_, CT_50/high_ and TPB; Table 1). Year effect was close to significance for *μ*_max_, with maximum performance decreasing slightly over the years (Table 1). Geographical location had no significant effect on any TPC parameter.

**Table 1:** Effect of multilocus sequence typing genotype, year of collection and location on strain thermal performance estimates

	Estimate	*SE*	*F*‐value	(*df*1, *df*2)	*p*‐value
*μ*_max_ (*n* = 49)					
Genotype					
C	1.091	0.012	16.8141	(3, 44)	<0.001
E	0.993	0.015			
G	1.044	0.029			
A&H	0.928	0.026			
Location					
Northern	*1.035*	*0.020*	*1.4126*	*(1, 43)*	*0.241*
Southern	*1.006*	*0.012*			
Year	−0.007	0.004	3.9550	(1, 44)	0.053
*T*_opt_ (*n *= 46)					
Genotype					
C	26.118	0.160	3.9975	(3, 42)	0.014
E	25.762	0.179			
G	24.922	0.357			
A&H	25.391	0.292			
Location					
Northern	*25.432*	*0.262*	*0.2627*	*(1, 41)*	*0.611*
Southern	*25.589*	*0.153*			
Year	−*0.013*	*0.049*	*0.0656*	*(1, 40)*	*0.799*
CT_low_ (*n* = 41)					
Genotype					
C	17.577	0.178	5.2715	(3, 37)	0.004
E	16.511	0.212			
G	17.028	0.397			
A&H	16.668	0.459			
Location					
Northern	*16.864*	*0.320*	*0.2220*	*(1, 35)*	*0.640*
Southern	*17.036*	*0.199*			
Year	*0.057*	*0.055*	*1.0523*	*(1, 36)*	*0.312*
CT_high_ (*n* = 49)					
Genotype					
C	31.570	0.167	7.3322	(3, 45)	<0.001
E	32.471	0.200			
G	31.107	0.400			
A&H	32.720	0.326			
Location					
Northern	*32.144*	*0.285*	*0.5236*	*(1, 44)*	*0.473*
Southern	*31.902*	*0.171*			
Year	*0.005*	*0.054*	*0.0095*	*(1, 43)*	*0.923*
TPB (*n* = 41)					
Genotype					
C	13.994	0.275	7.8427	(3, 37)	<0.001
E	15.970	0.328			
G	14.079	0.614			
A&H	15.437	0.709			
Location					
Northern	*15.080*	*0.496*	*0.4048*	*(1, 35)*	*0.529*
Southern	*14.721*	*0.308*			
Year	−*0.069*	*0.086*	*0.6543*	*(1, 36)*	*0.424*

Notes

Marginal means are reported for each level of the qualitative variables (Genotype, Location), and slope is reported for the continuous variable Year. The values reported in italics are the ones that were obtained in the last step before variables were removed during model selection. The number of strains (*n*) that we used in each model is reported next to the response variable name.

Location had a significant effect on PC2 coordinates, with northern strains exhibiting lower PC2 values (Table 2), corresponding to cold adaptation (Figure 3). MLST genotype had a significant effect on PC3 coordinates (negative correlation between maximum performance and cold tolerance, Figure 3).

**Table 2:** Effect of multilocus sequence typing genotype, year of collection and location on strain coordinates along PCs

	Estimate	*SE*	*F*‐value	(*df*1, *df*2)	*p*‐value
PC1 (*n* = 49)					
Genotype					
C	−*0.006*	*0.040*	*1.3967*	*(3, 45)*	*0.256*
E	*0.063*	*0.048*			
G	−*0.123*	*0.097*			
A&H	*0.091*	*0.079*			
Location					
Northern	*0.092*	*0.068*	*2.1427*	*(1, 44)*	*0.150*
Southern	−*0.025*	*0.041*			
Year	−*0.005*	*0.013*	*0.1339*	*(1, 43)*	*0.716*
PC2 (*n* = 49)					
Genotype					
C	−*0.030*	*0.040*	*0.8276*	*(3, 43)*	*0.486*
E	−*0.037*	*0.059*			
G	−*0.146*	*0.094*			
A&H	*0.038*	*0.080*			
Location					
Northern	−0.094	0.039	6.1395	(1, 47)	0.017
Southern	0.030	0.031			
Year	*0.009*	*0.010*	*0.8898*	*(1, 46)*	*0.350*
PC3 (*n* = 49)					
Genotype					
C	−0.060	0.018	9.8473	(3, 45)	<0.001
E	0.051	0.021			
G	0.002	0.042			
A&H	0.120	0.034			
Location					
Northern	−*0.005*	*0.030*	*1.6892*	*(1, 44)*	*0.200*
Southern	*0.040*	*0.018*			
Year	*0.006*	*0.006*	*1.2330*	*(1, 43)*	*0.273*

Notes

Marginal means are reported for each level of the qualitative variables (Genotype, Location), and slope is reported for the continuous variable Year. The values reported in italics are the ones that were obtained in the last step before variables were removed during model selection. The number of strains (*n*) that we used in each model is reported next to the response variable name.

### 3.4 Determinants of virulence

**Figure 4:**
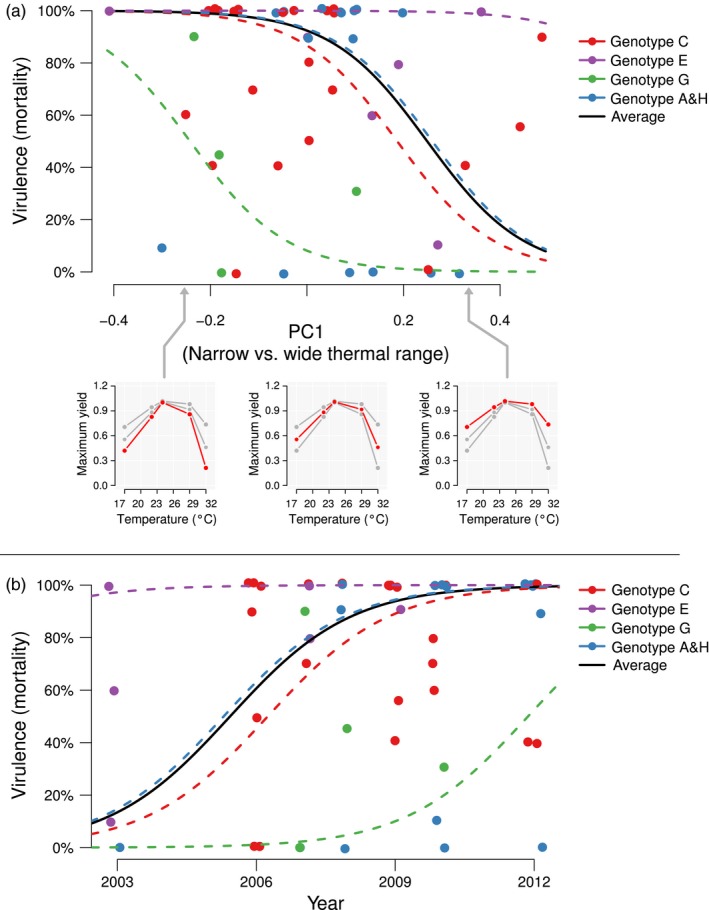
Effect of PC1 coordinate and year of collection on strain virulence. Each marker represents the average mortality observed for a given strain. Fitted curves are plotted using the GLMM results presented in Table 3 (*n* = 49 strains). Colours represent genotype groups. The black fitted line corresponds weight‐averaged fixed effect estimates based on the abundance of genotype groups in our dataset. Panel A, effect of PC1 coordinate on strain virulence. The three subpanels illustrate how TPC varies from specialist to generalist phenotype along PC1. Panel B, effect of year of collection on strain virulence

When the effect of thermal performance on virulence was analysed using TPC parameters estimated from curve fitting, 46 strains of 49 could be included in the analysis due to missing values in *T*_opt_. Year of isolation had a positive effect on virulence (Figure 4b, Table 3).

**Table 3:** Effect of strain characteristics on virulence (using PCs coordinates)

(*n* = 49 strains)	Estimate	*SE*	Chi‐square	*df*	*p*‐value
Genotype					
C	*P*_death_ * = *0.844	0.155	7.1673	3	0.067
E	*P*_death_ * = *0.918	0.121			
G	*P*_death_ * = *0.069	0.149			
A&H	*P*_death_ * = *1.000	0.001			
Location					
Northern	*P*_death_ * = 0.835*	*0.234*	*0.2283*	*1*	*0.633*
Southern	*P*_death_ * = 0.932*	*0.075*			
Year	1.962	0.869	**5.0903**	**1**	**0.024**
PC1	−1.960	0.872	**5.0560**	**1**	**0.025**
PC2	−1.373	0.859	2.5552	1	0.110
PC3	−1.516	0.964	2.4701	1	0.116

Notes

Model used in R: death ~ genotype + year + location + PC1 + PC2 + PC3 + (1|strain), with a binomial family distribution and a logit link function (*n* = 49 strains). Continuous variables (Year, PC1, PC2, PC3) were *z*‐normalized. Marginal means and standard errors are reported for each different level of qualitative variables (Genotype and Location) in the original response scale (*P*_death_), while slope estimates and standard errors in the logit scale are reported for the *z*‐normalized continuous variables. The values reported in italics are the ones that were obtained in the last step before variables were removed during model selection. Results with *p*‐values below 0.05 are highlighted in a bold font.

Among analysed TPC parameters, both *T*_opt_ and CT_50/high_ had an effect on virulence (Table 4): Strains with higher *T*_opt_ were more virulent and strains with higher tolerance to high temperatures were less virulent. When the effect of thermal performance on virulence was analysed using PCs (49 strains used), both year and PC1 coordinate had a significant effect on virulence: Strains collected more recently were more virulent (similarly as observed using TPCs) and strains with wider thermal range (PC1) had lower virulence (Table 4, Figure 4a).

**Table 4:** Effect of strain characteristics on virulence (using TPC estimates)

(*n* = 46 strains)	Estimate	*SE*	Chi‐square	*df*	*p*‐value
Genotype					
C	*P*_death_ * = *0.713	0.251	5.9872	3	0.112
E	*P*_death_ * = *0.869	0.163			
G	*P*_death_ * = *0.322	0.531			
A&H	*P*_death_ * = *1.000	0.001			
Location					
Northern	*P*_death_ * = 0.946*	*0.094*	*0.0153*	*1*	*0.901*
Southern	*P*_death_ * = 0.931*	*0.073*			
Year	1.962	0.954	**4.2277**	**1**	**0.040**
*μ*_max_	*0.689*	*1.079*	*0.4078*	*1*	*0.523*
*T*_opt_	2.168	0.902	**5.7709**	**1**	**0.016**
CT_high_	−2.459	1.069	5.2938	1	0.021

Notes

Model used in R: death ~ genotype + year + location + *μ*_max_ + *T*_opt_ + CT_50/high_ + (1|strain), with a binomial family distribution and a logit link function (*n* = 46 strains). Continuous variables (Year, *μ*_max_, *T*_opt_, CT_50/high_) were *z*‐normalized. Marginal means and standard errors are reported for each different level of qualitative variables (Genotype and Location) in the original response scale (*P*_death_), while slope estimates and standard errors in the logit scale are reported for the *z*‐normalized continuous variables. The values reported in italics are the ones that were obtained in the last step before variables were removed during model selection. Results with *p*‐values below 0.05 are highlighted in a bold font.

## 4 DISCUSSION

There is a growing body of evidence indicating that some pathogens become more prevalent (Chiaramonte, Munson, & Trushenski, 2016; Sterud et al., 2007) and more virulent at warmer temperatures (Smith et al., 2014). For example, it has been shown that increased expression of virulence factors is correlated with increased temperature in *Vibrio* species (Mahoney, Gerding, Jones, & Whistler, 2010; Oh, Lee, Lee, & Choi, 2009). In theory, virulence will evolve to a level at which virulence and transmission are balanced to optimize the spread of the pathogen (Alizon, Hurford, Mideo, & Van Baalen, 2009). Nevertheless, virulence is context–dependent, as both biotic factors such as host condition (Pulkkinen & Ebert, 2004) and host density (Bieger & Ebert, 2009) and abiotic factors such as temperature (Guijarro, Cascales, García‐Torrico, García‐Domínguez, & Méndez, 2015) can influence virulence. Consequently, pathogens that are able to survive outside their hosts and therefore less dependent on direct contact transmission are likely to experience nonoptimal virulence, high or low, depending on various factors that affects virulence outside the host (Bull & Ebert, 2008; Sundberg et al., 2014; Walther & Ewald, 2004). Temperature can have complex and even opposing effects on pathogens with free‐living stages and their ectothermic hosts, as high temperatures can cause stress and often lead to lowered host defences and increased susceptibility to infection, which could counteract the positive effects of temperature on abundance, transmission and better survival rates of the pathogen (Harvell et al., 2002).

In this study, we explored whether strains of an aquaculture‐associated pathogen vary in their thermal performance, and whether thermal performance was correlated with strain virulence. This type of information is crucial in predicting how climate change scenarios could alter environmental pathogens’ virulence via correlated selection on their thermal performance. To this end, we characterized the temperature dependency of maximum biomass yield of 49 isolates of *F. columnare* that were collected from eight different areas located across Finland between 2003 and 2012. We estimated their temperature performance curves (TPC) and used principal component analysis on raw performance measurements to assess the variation in thermal performance between strains. Our results revealed that despite northern location, Finnish *F. columnare* typically have a rather high optimum temperature between 23.7 and 27.9°C and an upper critical temperature for yield between 30.1 and 34.7°C. Finnish lakes form predominantly closed and shallow basins (average depth about 7 m) and surface waters may reach high temperatures in summer (Korhonen, 2002). As this bacterium can be isolated from natural waters (Kunttu, Sundberg, Pulkkinen, & Valtonen, 2012), tolerance to high temperature might be necessary for inhabiting natural waters during summer. Consistent with the idea that cold tolerance is a key element for survival and growth in high latitudes, isolates from Northern Finland were more tolerant to colder temperatures than isolates from southern Finland (see PC2 in Figure 3 and effect of location on PC2 in Table 2). Our findings are in agreement with previous studies showing that selection may favour higher performance in higher altitude/latitude environments to guarantee successful reproduction and transmission during short growth seasons (Yang et al., 2016). On the other hand, ample amount of genotype‐dependent variation in all TPC parameters (Table 1, Figure 2) suggests that genetic background might play a significant role in shaping thermal performance of this bacterium. These findings clearly indicate that thermal conditions can in principle have a strong effect on the genetic diversity of *F. columnar*e in the environment and therefore presumably also on disease dynamics.

Our results show that Finnish *F. columnare* strains have become more virulent in recent years, as evidenced in our experiments under controlled laboratory conditions where confounding effects such as increased environmental temperature, variable nutrient availability or variable host density were removed (year effect in Table 3 and Figure 4b) (see also Sundberg et al., 2016). Interestingly, not only did we find compelling evidence that higher optimum temperatures could be associated with increased bacterial virulence, but also bacterial virulence was negatively correlated with upper thermal tolerance (CT_50/high_) and with broader thermal performance curve (Tables 3 and 4). It has been shown in other fish pathogens that growth of bacteria at higher‐than‐optimal temperature can result in decreased virulence (Crosa, Hodges, & Schiewe, 1980; Ishiguro et al., 1981). This suggests that elevated temperatures or increased fluctuations associated with climate change should not select for higher virulence in this species. Nevertheless, the constraints of elevated temperature that we tested here (such as 25°C) on virulence should be relatively limited in temperate regions such as Finland. Therefore, there will be an opportunity for increasing virulence and more potential for deadly outbreaks in future Finnish aquaculture due to the following reason(s): (a) Ambient temperatures in the Finnish farming environment rarely exceed 25°C, (b) Guijarro et al. (2015) showed that some bacterial diseases in aquaculture, particularly those of freshwater fish, could occur at temperatures below bacterial optimal growth (i.e., optimum growth temperature for the fastest growth under laboratory conditions), (c) the summer water temperature in the fish farms could potentially be kept under relative control in some flow‐through farm systems due to the mixing of ground water with the inflow water from natural bodies. Yet, *F. columnare* occurs globally and negative association of virulence with CT_50/high_ provides relevant information for tropical environments where water temperature may exceed 30°C.

On the other hand, growth season and abundance of *F. columnare* is expected to increase as a result of the longer summer associated with climate warming, as temperature records from two fish farms in Northern and Central Finland over the past few decades show that the duration of the warm‐water season has increased (Supporting information Figure S3B). It has also been shown that the thickness of ice in Finnish lakes will decrease and the ice‐covered period will be considerably shorter than today (Elo, Huttula, Peltonen, & Virta, 1998). Therefore, the increase in the length of the pathogen growth season could allow for faster rate of adaptation in parasite traits under selective pressure during host exploitation (Supporting information Figure S3A and B). Consequently, coupled with intensive farming in Finland, these climate changes would further increase the severity of columnaris disease (Sundberg et al., 2016). Virulence in the wild is a complex interplay between host, parasite and environment, and future experimental studies should include variation in infection temperature to tease apart the role of temperature for both the bacteria and the host. This is especially important for salmonid fish (the natural hosts of columnaris disease) because they are cold‐adapted and increased stress due to high temperature may lead to higher morbidity.

We also found that maximum performance was overall negatively correlated with thermal performance breadth, suggesting a trade‐off between generalism and high‐performance specialism (negative correlation between *μ*_max_ and TPB in Figure 2). This supports to some extent the classic generalist–specialist trade‐off hypothesis. However, the main variation patterns found by PCA separate variation in thermal generalism (PC1) from variation in maximum performance (PC2 and PC3). Based on those two axes (PC2 and PC3, Figure 3), maximum performance is associated with CT50/low and CT50/high in complex ways. As TPB itself is defined by the difference between CT50/high and CT50/low, the overall trade‐off between *μ*_max_ and TPB observed in Figure 2 might be an indirect correlation resulting from the sum of those relationships. It is noteworthy that theories about specialism/generalism trade‐off are highly idealized and a “Jack of all temperatures” does not always have to be a master of none (Angilletta, 2009). Genotypes can have broader thermal performance range without always paying a visible performance cost at optimum conditions, but possibly involving a trade‐off with other traits (Huey & Hertz, 1984; Ketola et al., 2013), such as virulence (Ketola et al., 2013; Sturm et al., 2011). For environmentally growing opportunist pathogens, adaptations for more efficient exploitation of one growth environment could be expected to cause repercussions in their ability to grow in the other environment (Brown et al., 2012), such as host environment. Alternatively, the presence of virulence factors in the bacteria is unnecessary during the planktonic state but essential for the infection process, helping bacteria to save energy by not expressing virulence genes until they sense they have entered the host environment (Guijarro et al., 2015). This could explain why more generalist strains with broader thermal performance breadth were less virulent than strains with narrower TPB (see: PC1 effect in Table 3 and Figure 4a). Similarly, expression of virulence factors was found to decrease outside‐host growth rate in *Salmonella typhimurium* (Sturm et al., 2011) and adaptation to tolerate thermal fluctuations and protozoan predators have caused lowered virulence in experimental evolution settings with microbial pathogens (Friman et al., 2011; Ketola et al., 2013; Mikonranta et al., 2012; Zhang et al., 2014).

In conclusion, it seems that current problems with steadily increased severity of outbreaks and evolved virulence cannot be directly linked to increased mean temperature at fish farms and associated bacterial evolution. Still, the clear genotype and location effects on several thermal tolerance parameters suggest that temperatures might play strong role in dictating diversity and geographical distribution of this important fish pathogen.

## CONFLICT OF INTEREST

None declared.

## DATA ARCHIVING

Data available from the Dryad Digital Repository: https://doi.org/10.5061/dryad.858j5m7
