# Supplementary figures and images for: Broad thermal tolerance is negatively correlated with virulence in an opportunistic bacterial pathogen
Source: Evol Appl. 2018 Jul 11;11(9):1700–14. doi: 10.1111/eva.12673 (PMC6183471; doi:10.1111/eva.12673)

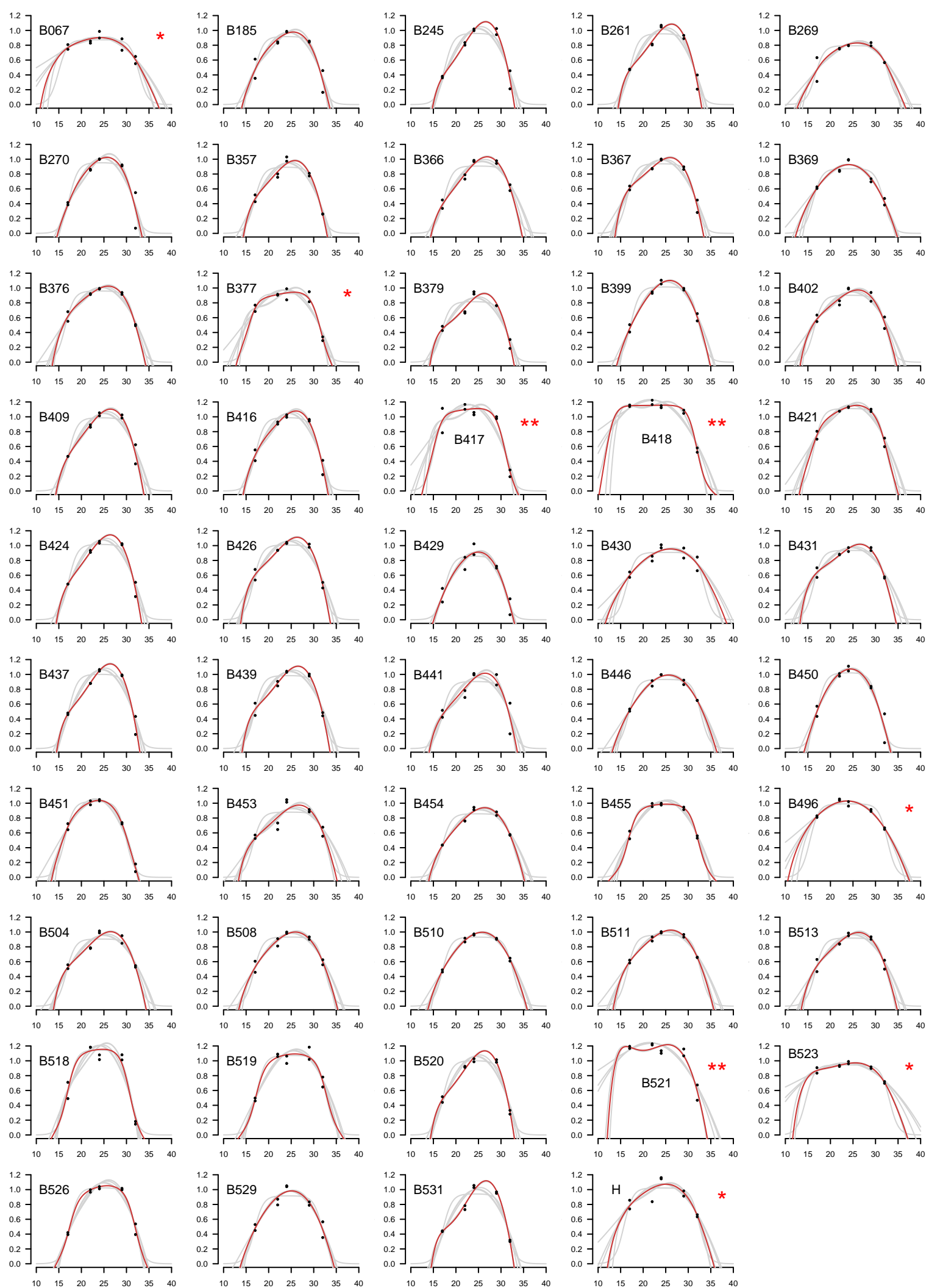

Supplement: Supplementary file 1 [file EVA-11-1700-s001.pdf]

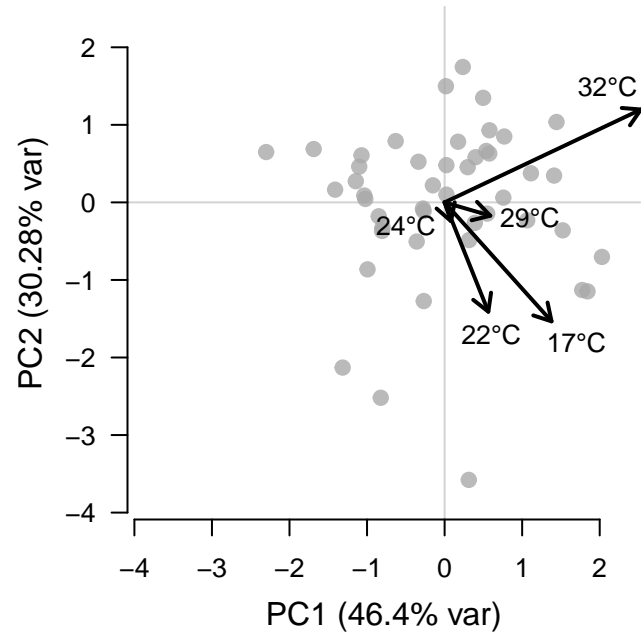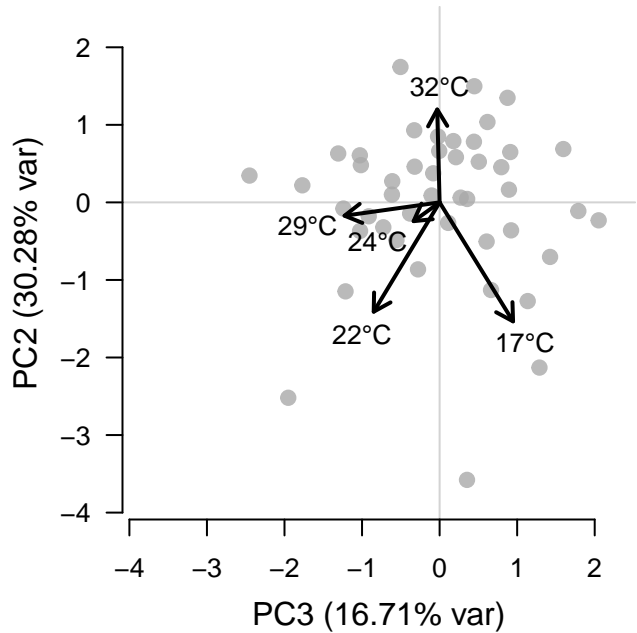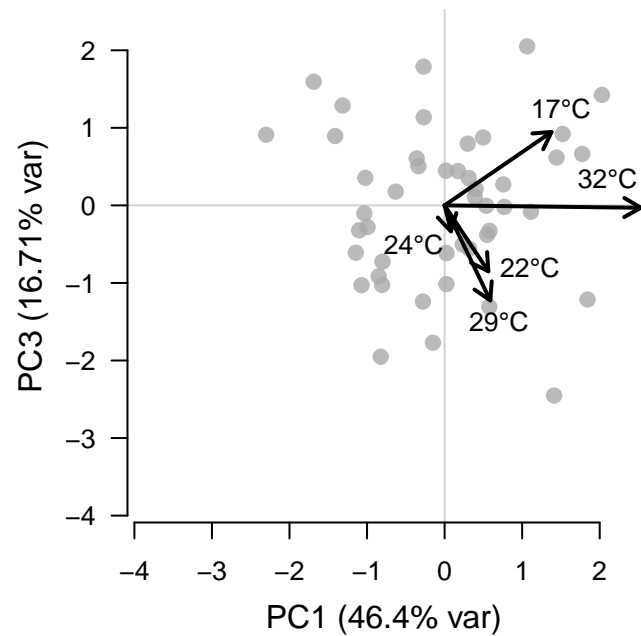

Supplement: Supplementary file 2 [file EVA-11-1700-s002.pdf]

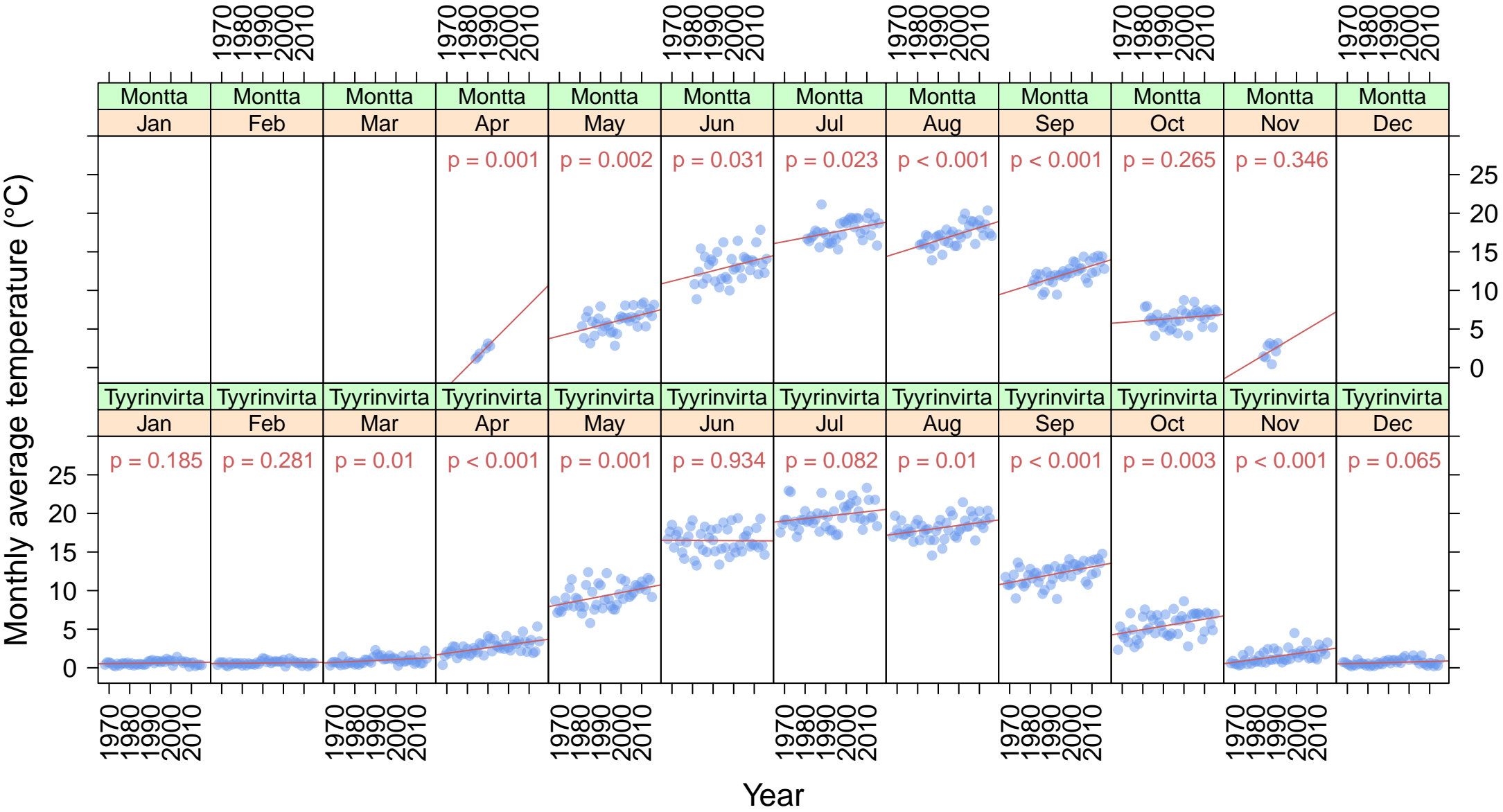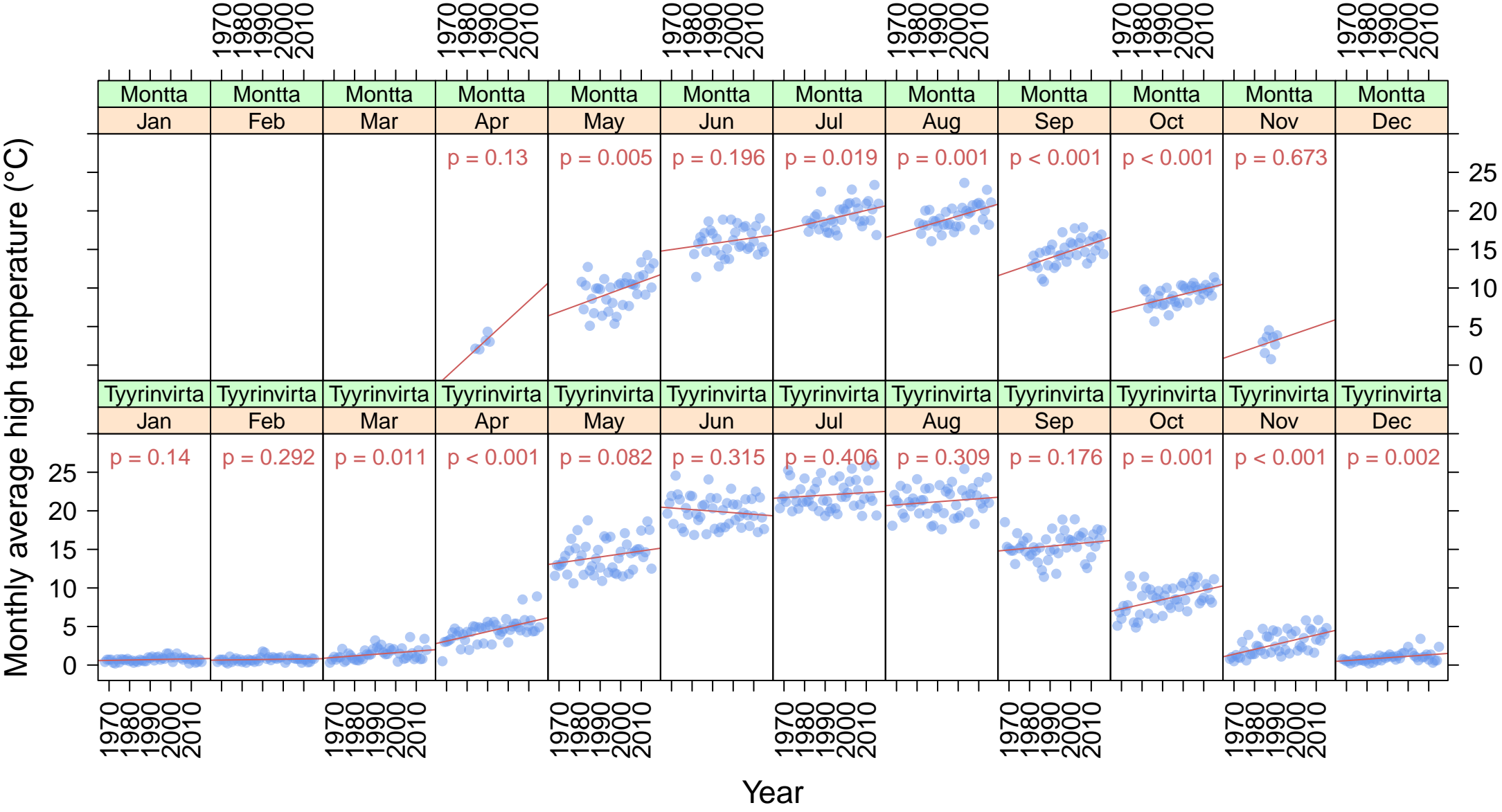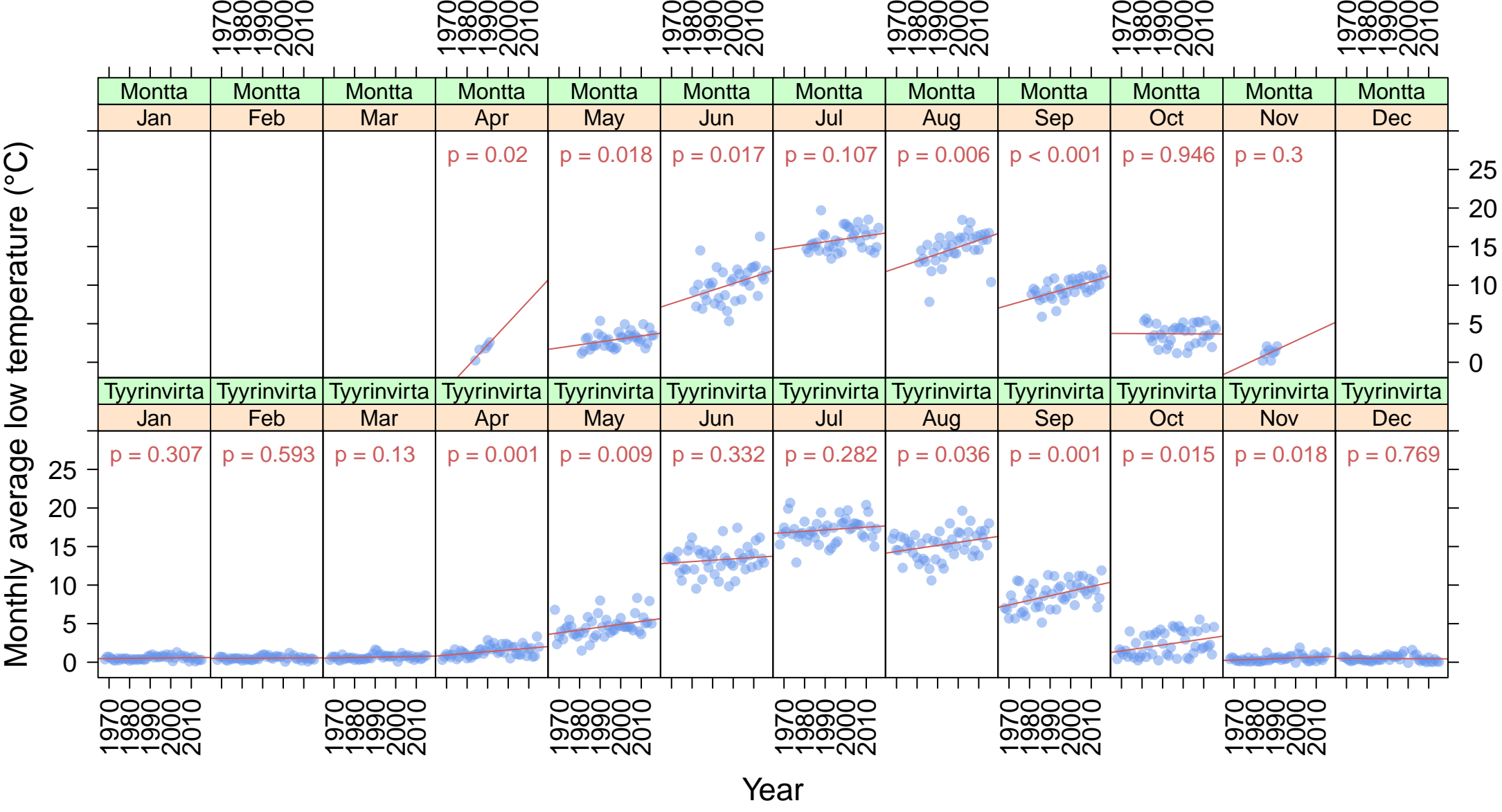

Supplement: Supplementary file 3 [file EVA-11-1700-s003.pdf]

### Montta

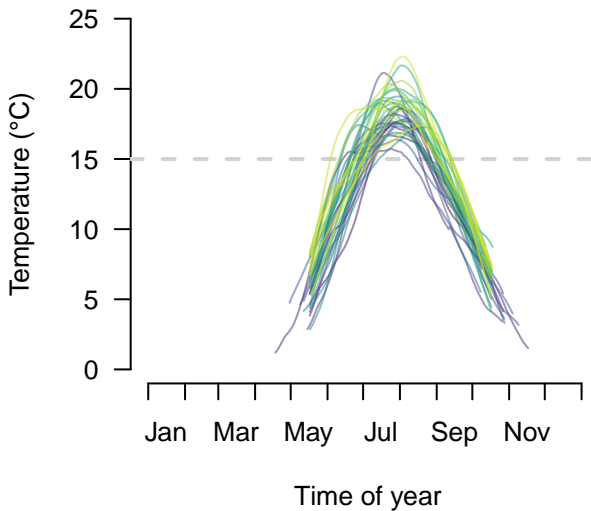

### Montta

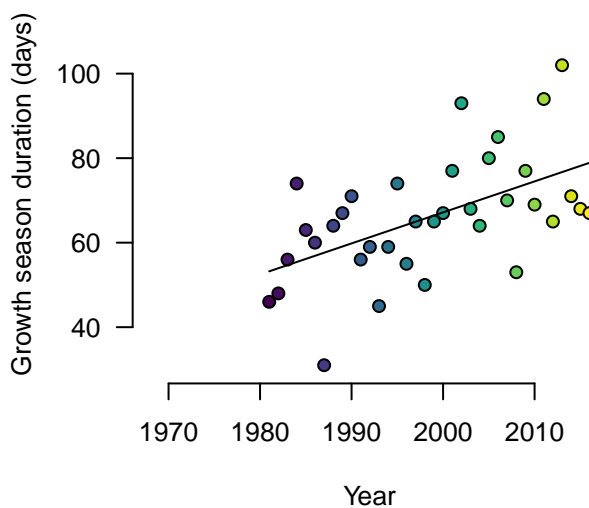

### Tyyrinvirta

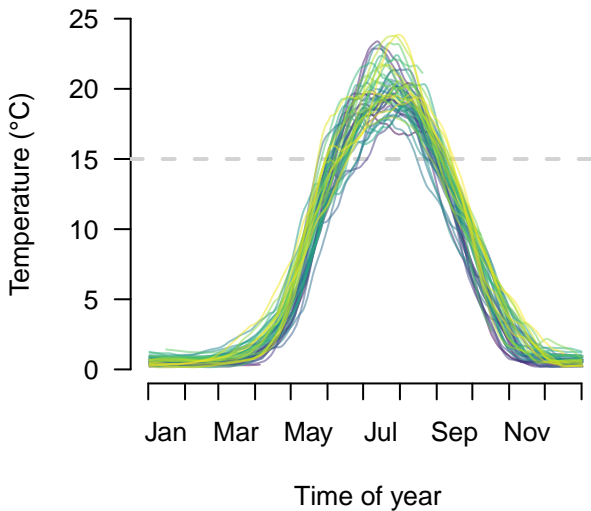

### Tyyrinvirta

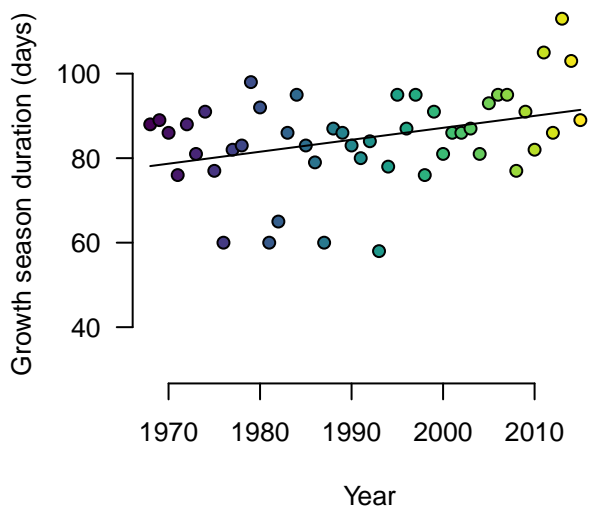

Supplement: Supplementary file 4 [file EVA-11-1700-s004.pdf]
